# Discrimination of Adulterated Ginkgo Biloba Products Based on 2T2D Correlation Spectroscopy in UV-Vis Range

**DOI:** 10.3390/molecules27020433

**Published:** 2022-01-10

**Authors:** Agata Walkowiak, Kacper Wnuk, Michał Cyrankiewicz, Bogumiła Kupcewicz

**Affiliations:** 1 Department of Inorganic and Analytical Chemistry, Collegium Medicum in Bydgoszcz, Nicolaus Copernicus University in Torun, 85-089 Bydgoszcz, Poland; agatawalkowiak1991@gmail.com; 2 Department of Biostatistics and Biomedical Systems Theory, Collegium Medicum in Bydgoszcz, Nicolaus Copernicus University in Torun, 85-067 Bydgoszcz, Poland; kacper.wnuk@cm.umk.pl; 3 Department of Biophysics, Collegium Medicum in Bydgoszcz, Nicolaus Copernicus University in Torun, 85-067 Bydgoszcz, Poland; micy@cm.umk.pl

**Keywords:** UV-Vis spectroscopy, two-trace two-dimensional spectroscopy, adulteration, Ginkgo biloba, food supplements

## Abstract

*Ginkgo biloba* is a popular medicinal plant widely used in numerous herbal products, including food supplements. Due to its popularity and growing economic value, G. biloba leaf extract has become the target of economically motivated adulterations. There are many reports about the poor quality of ginkgo products and their adulteration, mainly by adding flavonols, flavonol glycosides, or extracts from other plants. In this work, we developed an approach using two-trace two-dimensional correlation spectroscopy (2T2D COS) in UV-Vis range combined with multilinear principal component analysis (MPCA) to detect potential adulteration of twenty G. biloba food supplements. UV-Vis spectral data are obtained for 80% methanol and aqueous extracts in the range of 245–410 nm. Three series of two-dimensional correlation spectra were interpreted by visual inspection and using MPCA. The proposed relatively quick and straightforward approach successfully differentiated supplements adulterated with rutin or those lacking ginkgo leaf extract. Supporting information about adulteration was obtained from the difference between the DPPH radical scavenging capacity of both extracts and from chromatographic (HPLC-DAD) fingerprints of methanolic samples.

## 1. Introduction

The medical use of Ginkgo biloba was first recorded in the 16th century, while in the 1960s, the effectiveness of ginkgo in peripheral circulatory disorders and cerebrovascular diseases was discovered [1]. Nowadays, *Ginkgo biloba* leaf extract is one of the commonly used herbal medicine that can be used in the treatment of many diseases, including cognitive impairment and Alzheimer’s disease [2,3], tinnitus [4], retinal disease [5], diabetic nephropathy [6], ischemia cerebrovascular [7], and cardiovascular diseases [8].

Among the natural ingredients of *Ginkgo biloba* are many flavonoids with a unique structure, ten diterpenoid lactones, ginkgolides (A, B, C, J, K, L, M, N, P, and Q), five classes of alkylphenols (cardanols, α-hydroxycardanols, cardols, urushiols, and izourushiols) and phenolic acids (protocatechuic acid, p-hydroxybenzoic acid, vanillic acid, isovanillic acids, caffeic acid, p-coumaric acid, ferulic acid, sinapic acid, gallic acid), lignans, proanthocyanidins, polyphenols, and polysaccharides, among others [1].

The pharmaceutical market offers a wide range of products with *Ginkgo biloba* leaf extract registered as herbal medicines or food (dietary) supplements. The standardized *G. biloba* leaf extracts should contain about 24% flavonoids, 6% terpene trilactones, and less than five ppm of ginkgolic acid [9,10]. Although all the commercial ginkgo leaf products should follow these standards, many economically motivated adulteration issues have been reported in food supplements containing *Ginkgo biloba* extract [11]. Among different ways of ginkgo extract adulteration is the use of flavonol aglycones (quercetin and kaempferol), glycoside (quercetin 3-rutinoside), or other plant extracts containing flavonols, for instance, *Sophora japonica* fruit or flower extract [9,12,13]. A reliable adulteration detection method is needed to ensure the high quality of food supplements containing *Ginkgo biloba* extract available on the market and eliminate products with undeclared components. The assessment of physical and chemical properties of samples with a complex chemical composition is undoubtedly one of the current chemical analysis challenges. Chromatographic methods, such as HPLC, provide essential information about *Ginkgo biloba* leaf extract. However, these methods are relatively expensive, time-consuming, and require a long time to prepare the sample for analysis. Currently, there is also a need for constant vigilance in the quality control of herbal products. Sometimes, recommended pharmacopeial methods do not detect adulterations, as with *Ginkgo biloba* extracts adulterated by flavonol aglycones [9]. In addition, the quality of the plant raw material often depends on many parameters and the overall composition of a chemical product; therefore, its assessment usually requires a global approach. An analysis or immediate sample identification (e.g., suspicion of forgery) needed a method with a short analysis time, inexpensive, and without long-term sample preparation. Spectroscopic and chromatographic techniques often provide characteristic chemical fingerprints, which are the source of information about the composition of the analyzed sample. Plant fingerprints can be crucial in identifying and classifying herb species, their origin, and even detecting contamination or adulteration. The plant fingerprint approach could become a promising, expected, and powerful tool for quality assessment [14,15].

The choice of fingerprinting techniques depends on the properties of the constituents of the plant material. One frequently used method is HPLC coupled with various detectors, such as UV and DAD [15]. Chromatographic fingerprints of herbal products are usually very complex and the separation of all active ingredients in one chromatographic run is often difficult, so the development of this type of fingerprint is demanding and time-consuming. However, chromatographic fingerprints are a well-accepted technique in the pharmacognosy and quality control of herbs and plants, including ginkgo biloba extracts [9,16,17,18].

An alternative to chromatographic techniques are spectroscopic methods, e.g., Fourier Transform Infrared Spectroscopy (FTIR), Near-Infrared Spectroscopy (NIR), Nuclear Magnetic Resonance (NMR), and UV-Vis spectroscopy. Spectroscopy in the UV-Vis range is a common and inexpensive technique. Some works have demonstrated the effectiveness of the UV-Vis technique combined with the chemometric methods to detect food adulteration. An example is the possibility of supporting official analytical methods by a promising screening approach using fluorescence or UV-Vis spectroscopy to authenticate or detect adulteration in extra virgin olive oil with cheaper, lower quality vegetable oils [19,20]. Another example is the use of UV-Vis spectroscopy supported by chemometrics to quantify the level of corn adulteration in peaberry specialty coffee with different bean processing methods [21]. UV-Vis spectral fingerprints combined with chemometric techniques (SIMCA, PLS-DA, and SVM) have successfully been used to control the quality of mints (peppermint and spearmint) [22]. Chai et al. [23] show that UV-Vis spectroscopy combined with principal component analysis (PCA) can distinguish two fish species accurately. The fish sample was boiled for 2 min in trifluoroacetic acid (TFA) to obtain the extract for UV-Vis spectroscopic analysis. The purpose of another work was to explore the ability of UV-Vis, NIR, and MIR spectroscopy combined with chemometric techniques for detecting adulteration of beef with turkey meat [24]. Using UV-Vis spectroscopy and chemometric techniques, other authors have presented a screening method to distinguish counterfeit and genuine white and rested tequila [25]. UV-Vis spectroscopy was also used for authentication and detection geographical origin of tea [26], Boletus mushrooms [27], and saffron (*Crocus sativus* L.) [28].

In addition to the spectroscopic methods that are the most common source of one-dimensional (1D) signals, using the two-dimensional correlation spectroscopy (2D COS) algorithm, it is possible to generate a two-dimensional (2D) spectral fingerprint. Compared with conventional 1D spectroscopy, 2D correlation spectroscopy can provide information about the relationships between the absorption peaks of various functional groups and effectively extract characteristic information about the weak, overlapping, and shifting bands. Therefore, 2D COS is suitable for discriminating similar samples that are difficult to distinguish by conventional 1D spectroscopic methods. Two-dimensional correlation spectroscopy continuously evolves and grows with new developments and successful applications in various scientific fields such as environmental, medical, and food safety [29]. For example, 2D correlation infrared spectroscopy with or without perturbation has already been successfully applied across multiple adulteration issues, such as detection of milk adulteration with melamine [30,31,32], detection of *Aquilariae Lignum Resinatum* adulteration [33,34], analysis of crystallized lactose in milk powder [35], and discrimination of authentic and counterfeit *Polyporus umbellatus* [36].

An interesting direction of development of 2D COS is a new idea called two-trace, two-dimensional correlation spectroscopy (2T2D COS), which was introduced by Noda [37] in 2018 and then developed and described in another work [38]. In this approach, only a pair of spectra are needed to obtain a correlation spectrum, and no perturbation is required. The recently proposed 2T2D analysis as a simple and alternative approach to 2D correlation has been successfully applied in various physicochemical studies. For example, 2T2D COS has been used to explain the variability at the molecular level of the polymer system (polyamide 6) induced by water absorption [39], to detect structural changes in surfaces during adsorption processes [40], and for the characterization of polymer reorientation [41]. A temperature-perturbed IR measurement combined with 2T2D COS has also been proposed to improve defect identification in a pre-coated painted metal panel. [42] 2T2D correlation analysis, effectively identifying asynchronous spectra behavior in voltage-induced SERS (Surface-enhanced Raman scattering) spectra, were used to improve discrimination between stone and polyp gall bladders [43]. Other works have shown that two-dimensional correlation spectroscopy is an effective tool to enhance the accuracy of the discriminant analysis of pure and adulterated samples of olive oil, the determination of the geographic origin of milk vetch root (MVR) and perilla seed samples [44]. Moreover, 2T2D COS helped understand the changes in the macromolecular composition and assess the quality of Spirulina and its food products [45]. Our previous work presented the potential application of mid-infrared spectroscopy with ATR sampling and 2T2D correlation spectroscopy to differentiate samples of Ginkgo biloba dietary supplements adulterated with various flavonols [46].

This work presents a potential application of the two-trace two-dimensional correlation spectroscopy (2T2D COS) based on UV-VIS spectra to identify *Ginkgo biloba* food supplements adulterated with three flavonols: rutin, quercetin, and kaempferol. UV-VIS absorption spectra were recorded for 80% methanolic and aqueous extracts of 20 food supplements commercially available on the Polish market. Interpretation of 2T2D correlation spectra was supported by a multivariate principal component analysis (MPCA).

## 2. Results and Discussion

### 2.1. Chromatographic Fingerprints

**Figure 1:**
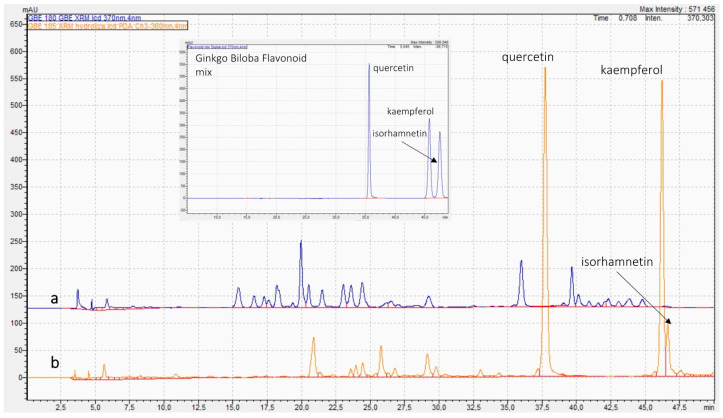
Chromatograms of *Ginkgo biloba* reference material (GB-XRM) pre-acidic hydrolysis (a) and post-acidic hydrolysis (b).

Chromatograms of unhydrolyzed samples (Figures S1 and S2) showed wide variability. Several products had large peaks corresponding to retention times of flavonols (rutin, quercetin, and kaempferol), indicating potential adulteration of these samples by the addition of flavonols. The presence of flavonols aglycones may also results from unique extraction process. The use of hydrochloric acid solution instead of recommended organic solvents (ethanol or acetone) in the manufacturing process of ginkgo extracts, provides the products with high amounts of aglycones (higher than their natural content). Figure 1 shows chromatograms of *Ginkgo biloba* reference material (GB-XRM) pre- and post-acidic hydrolysis. In *Ginkgo biloba* leaves, most of the flavonoids are flavonol glycosides, mainly quercetin, kaempferol, and isorhamnetin. After hydrolysis, the flavonoids are converted to respective aglycones. Figure 1 and Figure S3 clearly show this transformation. The post-acidic hydrolysis chromatogram revealed the highest intensities of peaks corresponding to quercetin and kaempferol, and the relatively more minor peak of isorhamnetin.

The large amount of rutin confirms its intentionally addition or use other rutin-reach plant extract, which means adulteration of ginkgo products. The presence of high peaks corresponding to quercetin and kaempferol may suggest both intentional adulteration or non-standard extraction process.

**Figure 2:**
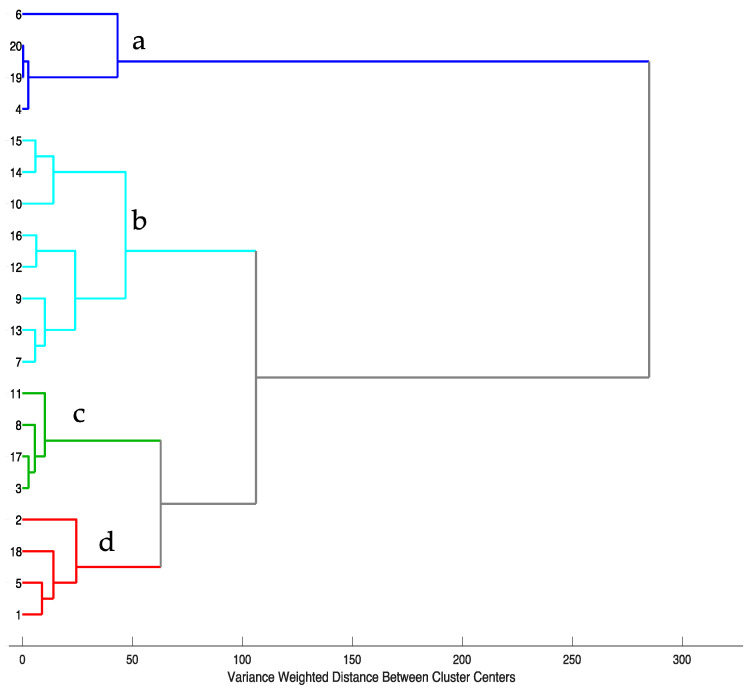
Hierarchical cluster analysis based on chromatographic data after mean center preprocessing. Ward’s method of agglomeration with Euclidean distance was used.

Figure 2 shows the result of a hierarchical cluster analysis of food supplements based on the data in Table 1. In this analysis, four clusters were identified. The first cluster (**a**) consists of S4, S6, S19, and S20, with rutin as the dominant component. Cluster **c** comprises supplements (S3, S8, S11, and S17) having a comparable level of quercetin and kaempferol (approximately 1:1). In contrast, cluster **b** contains the most numerous supplements with a high and prevailing quercetin content. Cluster **d** comprises supplements S1, S2, S5, and S18, which demonstrate a relatively low percentage of all flavonols without any dominant compound.

### 2.2. UV-Vis Spectra

**Figure 3:**
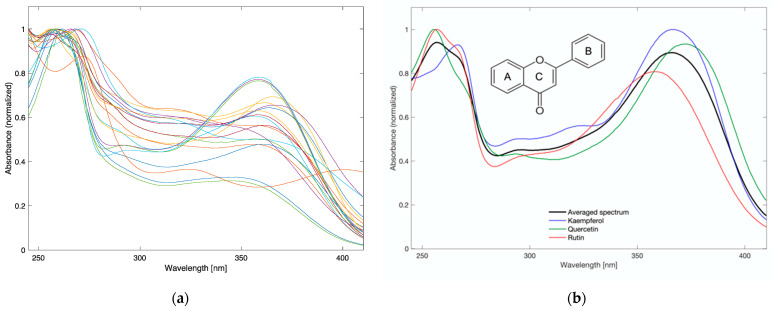
UV-Vis spectra of food supplements (**a**) and kaempferol, quercetin, and rutin (**b**) in 80% methanol.

Figure 3a shows UV-Vis spectra of methanolic extracts from food supplements and Figure 3b shows spectra of the three most common adulterants of the ginkgo biloba extract. Two aromatic rings (A and B) linked by heterocycle ring C form the basic flavonoid structure. In the UV-Vis region, flavonoids show two leading absorption bands: in the range 320–385 nm, which corresponds to ring B (cinnamoyl system, the band I), and in the range 240–280 nm, which corresponds to ring A (benzoyl system, band II) [16]. In the spectrum of rutin, absorption maxima are at 257 nm and 355 nm, quercetin has two bands with a maximum at 255 nm and 368 nm, and kaempferol shows the maximum absorption at 265 nm and 365 nm [16].

Most adulteration in ginkgo biloba extract can be detected by determination of the flavonoids profile. In raw ginkgo biloba leaf extract, the proportions of flavonol glycosides are stable, while the aglycones are present in trace amounts. Quercetin and kaempferol show relatively poor solubility in water and dissolve well in methanol. If quercetin, kaempferol, or rutin are artificially added to a dietary supplement, the bands characteristic for these compounds should appear in the UV-Vis spectrum of the methanolic solution. In contrast, they will be weaker or invisible (due to insufficient solubility) in the spectrum for the aqueous solution.

Additionally, to compare methanolic and aqueous extracts of samples determination of free radical scavenging capacity was determined. Detailed information about the methods and results can be found in the Supplementary Material, Section S2, Figure S2. Supplement samples containing an large amount of free aglycons (Table 1) show a significant difference in the value of antioxidant activity between the aqueous and methanol:water (80:20) extracts.

### 2.3. Two-Trace Two-Dimensional Correlation Spectroscopy (2T2D COS)

The UV-Vis spectra usually contain broad and relatively uncharacteristic bands. If tested samples have more than one UV-Vis absorbing component, they all contribute (additively) to the measured absorbance. Ginkgo products may contain “pure” standardized leaf extract or non-standardized extract, as well as adulterants including flavonols (kaempferol, rutin, and quercetin) or other plant extracts that are intrinsically multicomponent mixtures. The composition of ginkgo medical products is governed by strict guidelines, while food supplements may unpredictably differ from the manufacturer’s declaration. The presence of ginkgo extract and potential adulterants in the samples varies; however, the 2T2D correlation spectra are independent of the concentration of components in the sample. Therefore, we assumed that the correlation spectroscopy would allow us to visualize the differences in the spectra and obtain information about the presence of adulterants.

The basis of two-trace two-dimensional correlation spectroscopy were detailed described by Noda in [37,38]. The asynchronous spectra, antisymmetric along diagonal, indicate differences between correlated spectra. Cross peaks appear at the spectral coordinates between bands from both spectra originating from different molecules or compounds. We calculated three series of 2T2D asynchronous correlation spectra (according to Equation (2)) by compiling the UV-Vis spectra in different configurations. The first approach (series A) involves correlating spectra of 80% methanolic extracts of food supplements with water extracts spectra of those supplements. In series B, a spectrum of 80% methanolic extract from each food supplement was correlated with standard *Gingko biloba* leaf extract (GB-XRM) as a reference spectrum. In the third approach (series C), the reference was the averaged spectrum of potential adulterants, i.e., kaempferol, quercetin, and rutin.

**Figure 4:**
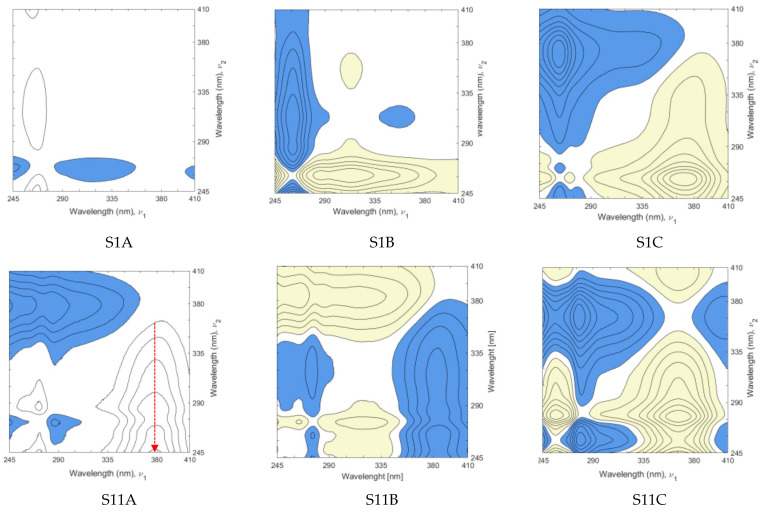

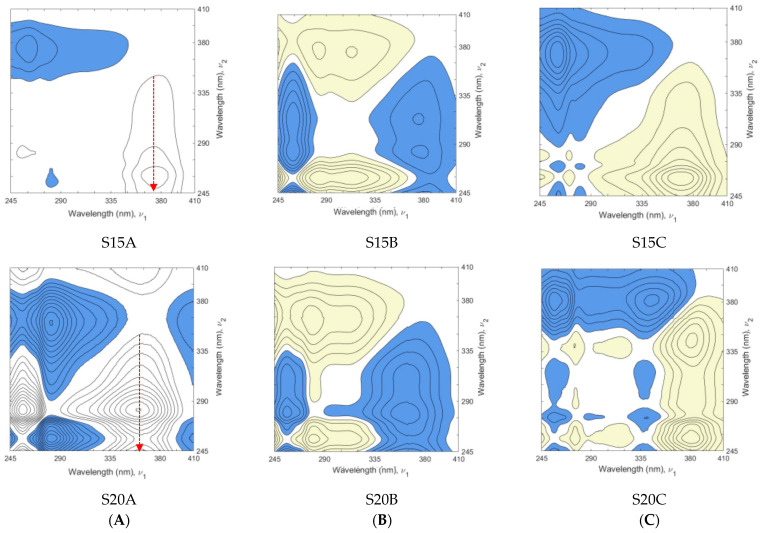
Representative asynchronous 2T2D correlation spectra: (**A**) spectra of 80% methanol extract vs. water extract, (**B**) spectra of food supplements vs. spectrum of ginkgo RM, and (**C**) spectra of food supplements vs. averaged spectrum of adulterants: kaempferol, quercetin, and rutin.

Figure 4 shows four exemplary 2T2D asynchronous correlation spectra (from series A, B, and C), representing the individual clusters in Figure 2. S15 (from cluster **b**) and S20 (included in cluster **a**), which are examples of supplements with quercetin and rutin as the dominant adulterant, respectively. S11 is an example of a supplement adulterated with quercetin and kaempferol (cluster **c**), while S1 represents cluster **d**. The other 2T2D asynchronous correlation spectra are shown in Figure S5 (Supplementary Material).

The first approach (series A) involves the correlation of the spectra of 80% methanol and aqueous extracts from each supplement. The presence of cross peaks in all spectra implies the differences between sample and reference spectrum due to the various solubility of the supplement components in methanol and water. The fewer cross peaks in the correlation spectrum and their smaller area indicate a marked similarity of the two extracts and correspond with the difference in DPPH radical deactivation (Figure S4). Additionally, the position of the cross peak (e.g., relative to the vertical axis; red arrow in Figure 4A) may suggest which adulterant is present in the supplement. For example, the position of the cross peak approximately at 360 nm for S20 demonstrates the presence of rutin. In correlation spectra of S11 and S15, the cross peak lies at ~372–375 nm, suggesting adulteration with quercetin or kaempferol, or both.

**Figure 5:**
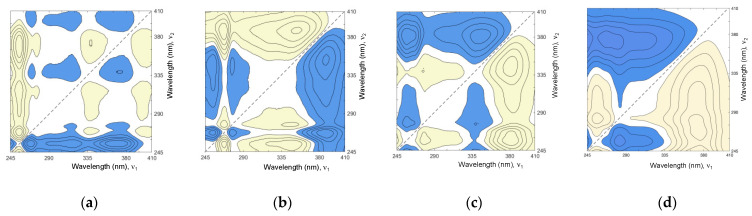
Asynchronous 2T2D correlation spectra of flavonoids kaempferol (**a**), quercetin (**b**), rutin (**c**), and ginkgo reference material (**d**) vs. the averaged spectrum of these three flavonoids.

The second series of 2T2D asynchronous correlation spectra (series B) was calculated using spectra of methanolic solutions of supplement and reference material of ginkgo leaf extract. UV-Vis spectrum of *G. biloba* leaf reference material is shown in Figure S6 (in Supplementary Material). In series C of asynchronous 2T2D spectra, the reference was the averaged spectrum of potential adulterants, i.e., kaempferol, quercetin, and rutin. The appearance of cross peaks in each spectrum originates from different source components. In the sample (food supplement), the presence of adulterant, e.g., kaempferol, should generate a unique set of cross peaks derived from the correlation between kaempferol in sample spectrum and rutin and quercetin in the reference spectrum (Figure 5a). The same applies to the presence of quercetin or rutin in a food supplement (Figure 5b,c). Figure 5d shows the correlation spectrum of ginkgo reference material vs. the averaged spectrum of kaempferol, quercetin, and rutin.

Table S1 shows the spectral coordinates of cross peaks visible on the plot (Figure 5) of asynchronous spectra of three adulterants and ginkgo RM correlated with the averaged spectrum of kaempferol, quercetin, and rutin. Although the sign of a cross peak may convey information about the intensity contribution of the moiety represented by the band in the sample spectrum or the reference spectrum, this is less important in such an approach. In contrast, the position and shape of the cross peaks provide valuable information relevant to the interpretation. Ginkgo extract contains many ingredients, including relatively small amounts of rutin, and is naturally free of kaempferol and quercetin (Figure 5d). Correlation of ginkgo RM with the averaged spectrum of adulterants results in two cross peaks, first at the spectral coordinates of 257/278 nm and the vast second cross peak with a characteristic spike.

### 2.4. Chemometric Analysis of 2T2D Correlation Spectra

**Figure 6:**
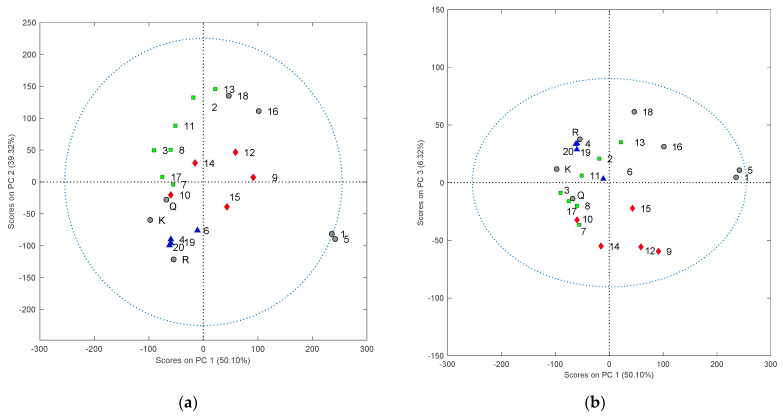
MPCA scores plot based on 2T2D correlation maps calculated for two spectra of 80% methanol extracts food supplements vs. ginkgo XRM as sample and reference spectrum, respectively. Figure (**a**) presents score plot PC1 vs. PC2 and (**b**) PC1 vs. PC3. RMSEC: 0.081; RMSECV: 0.101. The blue triangles indicate supplements with rutin as the main component, red diamonds indicate supplements with quercetin as the main component, and green squares represent supplements with quercetin and kaempferol as the main components. The probability of confidence ellipse was 90%.

Interpretation of asynchronous spectra in series B and C was supported with multilinear principal component analysis (MPCA). Figure 6 shows the scores of the first three principal components: PC1 vs. PC2 and PC1 vs. PC3 of all food supplement samples, using 2T2D correlation spectra in the range between 245 and 410 nm. The explained variance obtained for three PCs is 95.75% and values of RMSEC (Root Mean Square Error of Calibration) and RMSECV (Root Mean Square Error of Cross-validation) are 0.081 and 0.101, respectively. Additionally, Figure S8 presents a 3D scores plot and Figure S9a shows loadings plot.

According to information from chromatographic profiles, the selected supplements are marked with different colors representing three groups: products with rutin as the main component (blue), supplements with quercetin as the main component (red), and supplements with both quercetin and kaempferol as the main components (green). In Figure 6a, supplements S4, S19, S20, and S6 are close to each other and rutin (R), which shows their similarity. Supplements S1 and S5 are dissimilar from other supplements.

**Figure 7:**
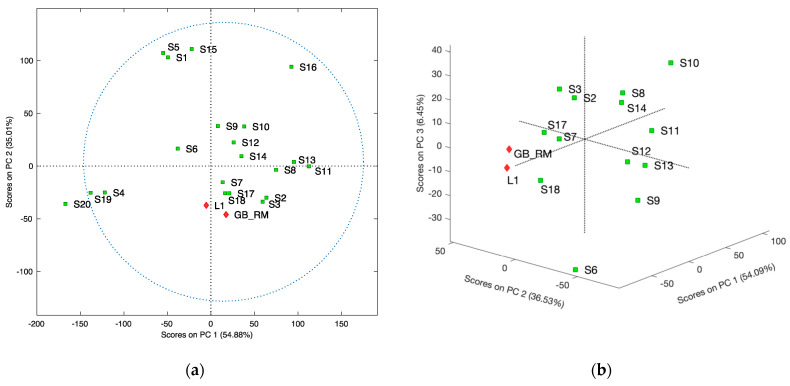
MPCA scores plot base on 2T2D correlation maps calculated for food supplements (sample spectra) vs. averaged spectrum of adulterants: kaempferol, quercetin, and rutin (reference spectrum), RMSEC: 0.066; RMSECV: 0.107; (**a**) plot including all samples; (**b**) MPCA scores plot after excluding samples S1, S4, S5, S15, S16, S19, and S20, RMSEC: 0.140; RMSECV: 0.264.

The results of MPCA analysis for the spectra of series C and two other correlation spectra: for the selected ginkgo medicinal product (L1) and reference material (GB_RM), which are presented in Figure 7 and Figure S9b. The first part (Figure 7a) shows the scores of the principal components PC1 vs. PC2. Supplements S4, S19, and S20 are close to each other what means their similarity. The second group consists of S1, S5, and S15, dissimilar from other supplements, and S16 is an outlier. The remaining objects belong to one relatively large cluster. To emphasize the similarity of the elements of this group, MPCA was again performed after excluding samples S1, S4, S5, S15, S16, S19, and S20. The results in Figure 7b revealed the similarity of S18 to L1 and GB-RM, both containing the standardized leaf extract of G. biloba. S18 is the only supplement that is free from adulterants.

For comparison, results of PCA based on conventional 1D UV-Vis spectra were shown in Figure S7.

## 3. Materials and Methods

### 3.1. Standards, Samples, and Reagents

Rutin, quercetin, and kaempferol were purchased from Sigma-Aldrich (Saint Louis, MO, USA). The solvents (acetonitrile, water, and ethanol) used in this study were all analytical and HPLC grade. In the survey, 23 herbal products containing Ginkgo biloba extract were analyzed. Three of them were herbal drugs with standardized (24/6) ginkgo extracts (one in the form of capsules and two in the form of tablets): Bilobil^®^ (Krka, Novo Mesto, Slovenia), Tanakan^®^ (Ipsen, Paris, France), and Tebokan^®^ (Dr. Willmar Schwabe GmbH & Co. KG, Karlsruhe, Germany). Reference material Ginkgo (Ginkgo biloba) Leaf XRM was purchased from Chromadex^®^ (Los Angeles, CA, USA). Ginkgo Biloba Flavonoid Mix (quercetin, kaempferol, and isorhamnetin) was purchased from Merck Supelco™. The other samples were food supplements. The commercial products were bought from local pharmacies and markets (Bydgoszcz, Poland) and online pharmacies. Products were coded as L1 to L3 (for medicinal products) and S1 to S20 (for herbal food supplements) to maintain the confidentiality of the supplier’s identity.

### 3.2. UV-Vis Spectroscopy

Two extracts for each drug and food supplement sample were prepared, 80% methanol and aqueous extracts, then filtered and adequately diluted. Shimadzu model UV-VIS spectrophotometer equipped with a quartz cell (10 mm optical path) was employed for the spectral measurements. For each sample, the measurement was carried out for the methanol and aqueous extract. The spectrum was registered in the range between 245 and 410 nm with a 1 nm resolution. The adjustment of the transmittance signal was performed using the same water or 80% methanol, used in the extraction step as blank.

### 3.3. Two-Trace Two-Dimensional Correlation Spectroscopy (2T2D COS) and Chemometric Methods

According to Noda [31], a pair of two spectra may be compared by constructing the synchronous $\Phi \left(\nu_{1},\nu_{2}\right)$ and asynchronous map $\Psi \left(\nu_{1},\nu_{2}\right)$:

(1) $$\Phi \left(\nu_{1},\nu_{2}\right)=\frac{1}{2}\left[s\left(\nu_{1}\right)\cdot s\left(\nu_{2}\right)+r\left(\nu_{1}\right)\cdot r\left(\nu_{2}\right)\right],$$

(2) $$\Psi \left(\nu_{1},\nu_{2}\right)=\frac{1}{2}\left[s\left(\nu_{1}\right)\cdot r\left(\nu_{2}\right)-r\left(\nu_{1}\right)\cdot s\left(\nu_{2}\right)\right]$$

where $s\left(\nu_{1}\right)$ is the sample spectrum and $r\left(\nu_{1}\right)$ is the reference spectrum.

The correlation spectra were preprocessed before chemometric analysis using normalization and autoscaling (with the scale offset 1 × 10^−5^). Cross-validation was employed by the venetian blinds method with ten splits and two samples per blind. All the calculations were performed using PLS-Toolbox 7.5 (Eigenvector Research, Inc., Manson, WA, USA) and MATLAB software version R2020b (The MathWorks, Inc., Natick, MA, USA).

### 3.4. Chromatographic Fingerprint Analysis

The reverse-phase high-performance liquid chromatography (RP-HPLC) was applied as a reference method. To obtain fingerprint chromatograms of commercial products with ginkgo extract, a Grace Smart^®^ column (150 mm x 4.6 mm, 5 µm) was used. The analysis was conducted using a high-performance liquid chromatograph with a photodiode array detector (HPLC-DAD, Shimadzu Corp., Kyoto, Japan). The mobile phase consisted of water with 0.2% formic acid (phase A) and acetonitrile with 0.2% formic acid (phase B). At a flow rate of 0.6 mL/min, the gradient was as follows: 0–4 min 17% B, 10–25 min 23% B, 30–37 min 30% B, 40–45 min 17% B. Detection was at a wavelength of 360 nm, and an injection volume of 5 μL was applied. Injection of each sample was performed in triplicate.

## 4. Conclusions

In summary, the proposed approach includes: (i) side-by-side preparation of two extracts of the food supplement with 80% methanol and water, (ii) registration of absorption spectra in the range of 245–410 nm, (iii) calculation of asynchronous correlation spectra according to Noda’s equation, (iv) examination of three-dimensional spectral datasets (correlation spectra) using MPCA. This approach may be a screening and qualitative tool to detect the presence or absence of adulterants in Ginkgo biloba leaf extract. The application of 2T2D correlation spectroscopy based on the UV-Vis spectra allows to detect and distinguish three groups of dietary supplements: (1) with the addition of rutin, (2) those containing a different extract (probably from a different plant), and (3) containing kaempferol or quercetin (as potentially adulterants or result of destructive extraction process). Distinguishing between supplements with adulterants quercetin and kaempferol, or both, is difficult due to the similarity of their spectra. Nineteen out of twenty tested food supplements contained large amounts of free aglycons. The results of our study suggest the vital need for more effective and stricter control of such products.

## Figures and Tables

**Table 1:** Percentage contribution of the rutin (R), quercetin (Q), and kaempferol (K) peak area in the total chromatogram area for food supplements.

Food Supplement	%R	%Q	%K
S1	11.67	5.05	9.62
S2	5.73	6.44	24.88
S3	4.03	29.13	21.36
S4	87.42	1.88	0.00
S5	3.16	3.96	11.36
S6	58.43	5.20	10.95
S7	3.99	26.91	13.80
S8	3.16	29.39	24.92
S9	5.51	24.38	3.07
S10	4.88	57.85	3.09
S11	8.77	30.98	28.64
S12	4.24	37.44	4.80
S13	4.33	22.85	9.76
S14	4.39	44.74	1.62
S15	6.26	48.57	2.30
S16	6.34	36.38	2.62
S17	2.95	28.35	18.93
S18	3.17	0.76	0.00
S19	86.52	0.00	0.00
S20	86.77	0.00	0.00

## Data Availability

Not applicable.

## Supplementary Materials

The following supporting information can be downloaded. Figure S1: Fingerprint chromatograms of 80% methanolic extracts of food supplements. Figure S2: Chromatograms of flavonoids standard mixture (a), methanolic extract of food supplement S18 (b), and methanolic extract of ginkgo medicinal product L1 (c). Figure S3: Chromatograms of Ginkgo biloba medicinal product before hydrolysis (a) and after acidic hydrolysis (b). Figure S4: The absolute difference between percentage inhibition of DPPH solution by 80% methanol and aqueous extracts of ginkgo preparations (a), averaged percentage of inhibition of DPPH solution by 80% methanol and aqueous extracts of ginkgo products. L1–L3 are medicinal products, S1–S20 food supplements, and GB-XRM is the Ginkgo biloba leaf extract reference material. Figure S5: Asynchronous 2T2D correlation spectra: (A) spectra of 80% methanol extract vs. water extract, (B) spectra of food supplements vs. spectrum of ginkgo RM, (C) spectra of food supplements vs. averaged spectrum of the adulterants: kaempferol, quercetin, and rutin. Figure S6: UV-Vis spectrum of Ginkgo biloba leaf extract (Reference Material) in 80% methanolic solution. Figure S7: PCA scores plots (a) and loadings plot (b) based on conventional 1D UV-Vis spectra of 80% methanolic extracts, RMSEC: 0.225; RMSECV: 0.596. L—ginkgo medicinal product, S1–S20—food supplements, GB-RM—Ginkgo biloba leaf extract (Reference Material). Figure S8: MPCA scores plot (PC1, PC2, and PC3) based on 2T2D correlation maps calculated for two spectra of 80% methanol extracts food supplements vs. ginkgo XRM as sample and reference spectrum, respectively. RMSEC: 0.081; RMSECV: 0.101. The blue triangles indicate supplements with rutin as the main component, red diamonds indicate supplements with quercetin as the main component, and green squares represent supplements with quercetin and kaempferol as the main components. The probability of confidence ellipse was 90%. Figure S9: Loadings plots for MPCA models based on spectra of series B(a) and series C(b). Table S1: The spectral coordinates of characteristic cross peaks in Figure 5 (s—sample spectrum, r—reference spectrum), + or − indicate positive or negative peaks.
