# Supplementary material for: Discrimination of Adulterated Ginkgo Biloba Products Based on 2T2D Correlation Spectroscopy in UV-Vis Range
Source: Molecules. 2022 Jan 10;27(2):433. doi: 10.3390/molecules27020433 (PMC8777600; doi:10.3390/molecules27020433)
Supplement: Supplementary file 1 [file molecules-27-00433-s001.zip › molecules-1497179-supplementary.pdf]

Discrimination of Adulterated Ginkgo Biloba Products Based on 2T2D Correlation Spectroscopy in UV-Vis Range

Agata Walkowiak[1], Kacper Wnuk [2], Michał Cyrankiewicz[3] and Bogumiła Kupcewicz [1,*]

[1] Department of Inorganic and Analytical Chemistry, Collegium Medicum in Bydgoszcz, Nicolaus Copernicus University in Torun, Poland; agatawalkowiak1991@gmail.com
[2] Department of Biostatistics and Biomedical Systems Theory, Collegium Medicum in Bydgoszcz, Nicolaus Copernicus University in Torun, Poland; kacper.wnuk@cm.umk.pl
[3] Department of Biophysics, Collegium Medicum in Bydgoszcz, Nicolaus Copernicus University in Torun, Poland ; micy@cm.umk.pl

*Correspondence: kupcewicz@cm.umk.pl

1. Chromathographic analysis

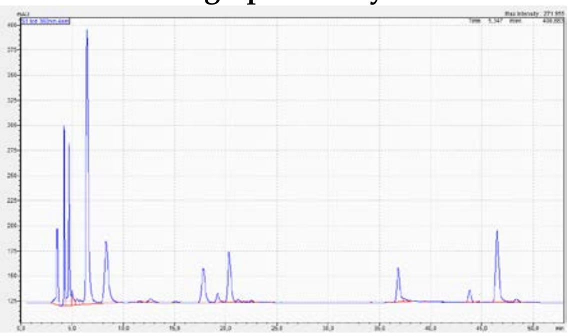

S1

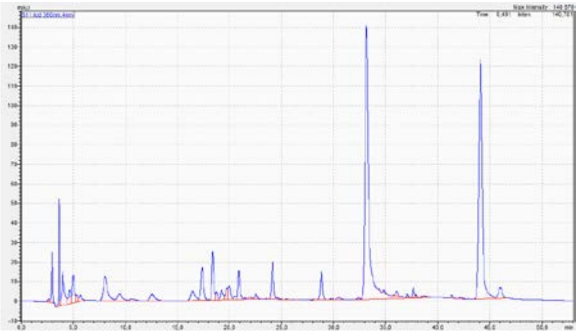

S11

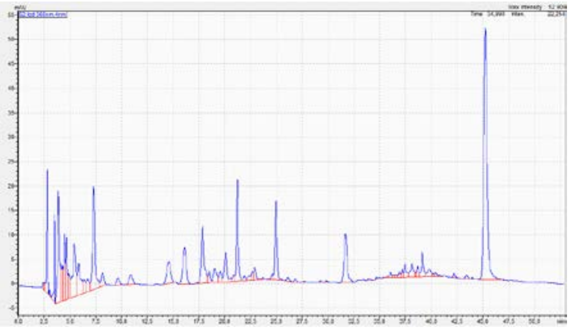

S2

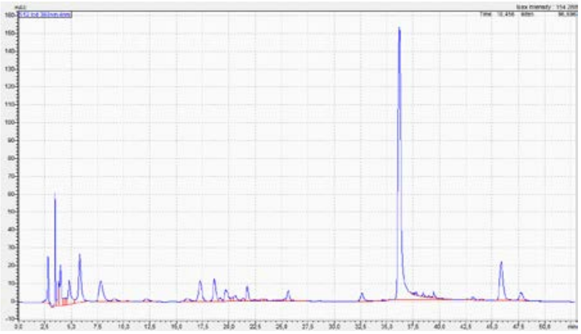

S12

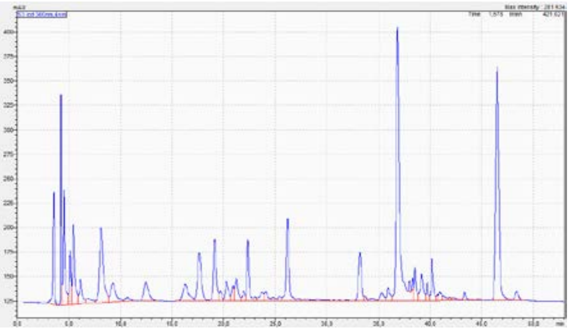

S3

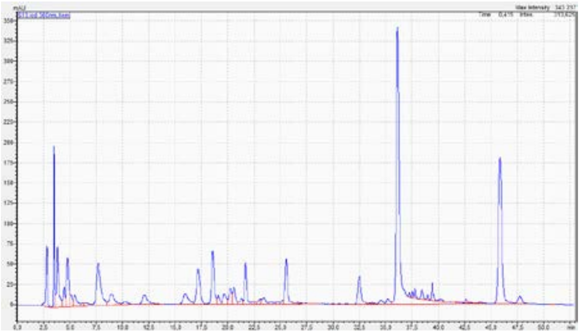

S13

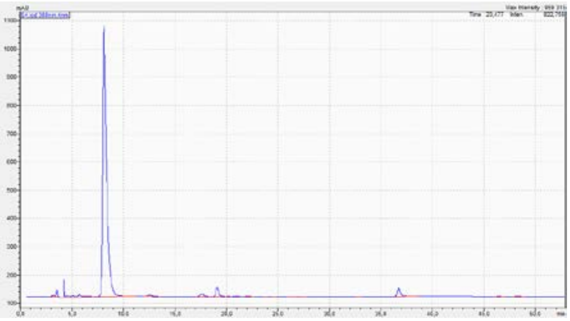

S4

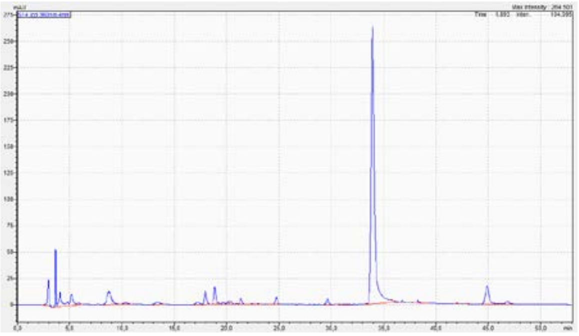

S14

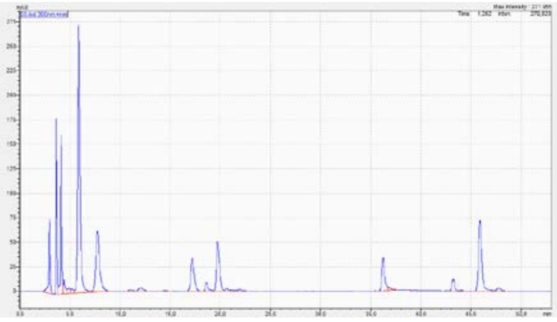

S5

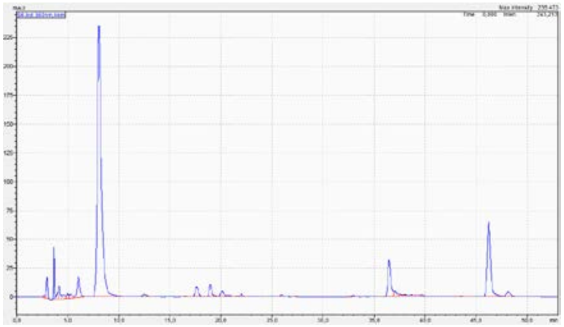

S6

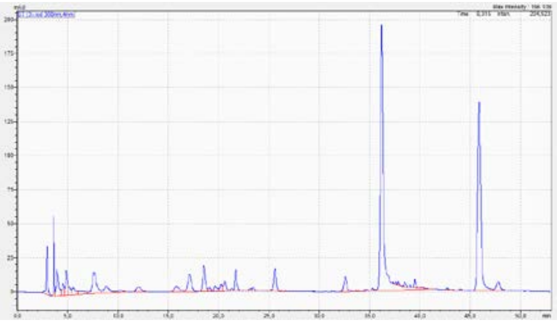

S7

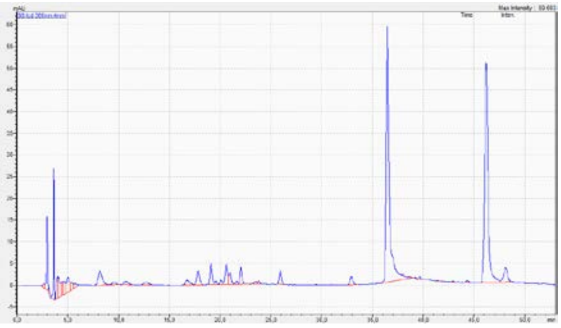

S8

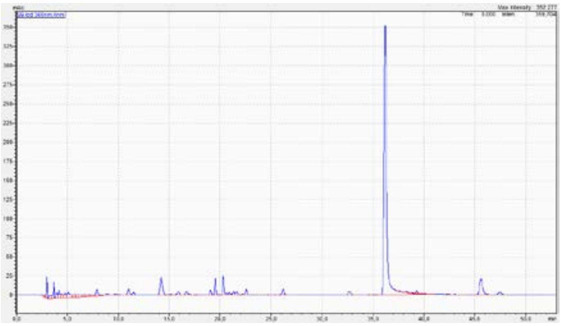

S9

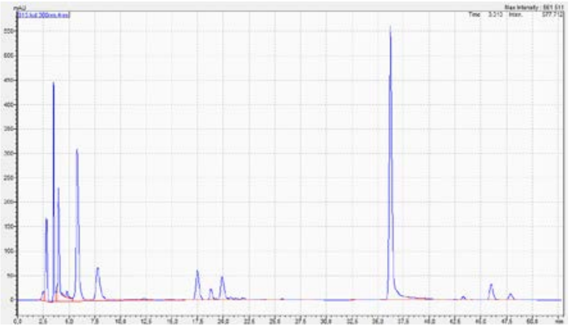

S15

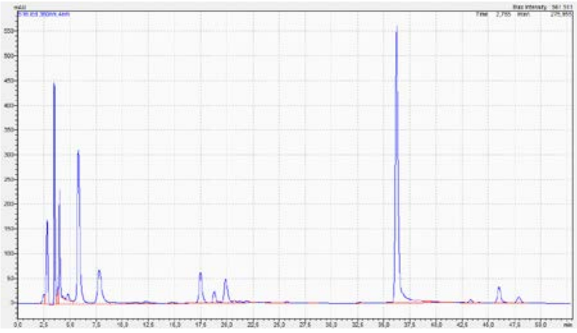

S16

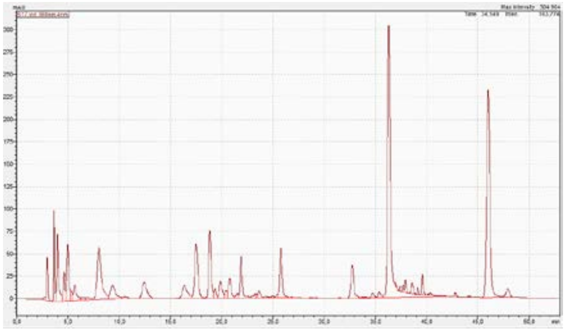

S17

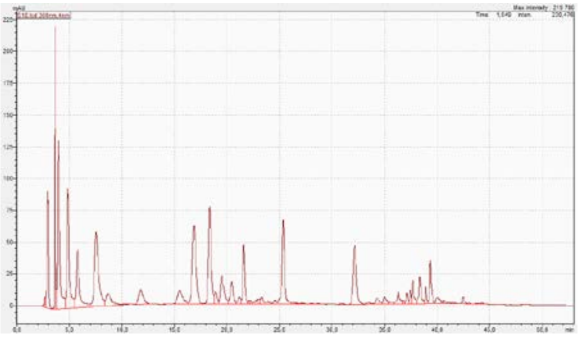

S18

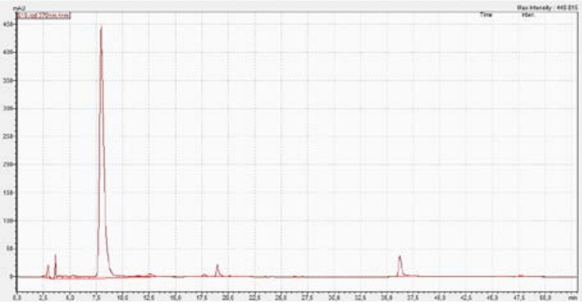

S19

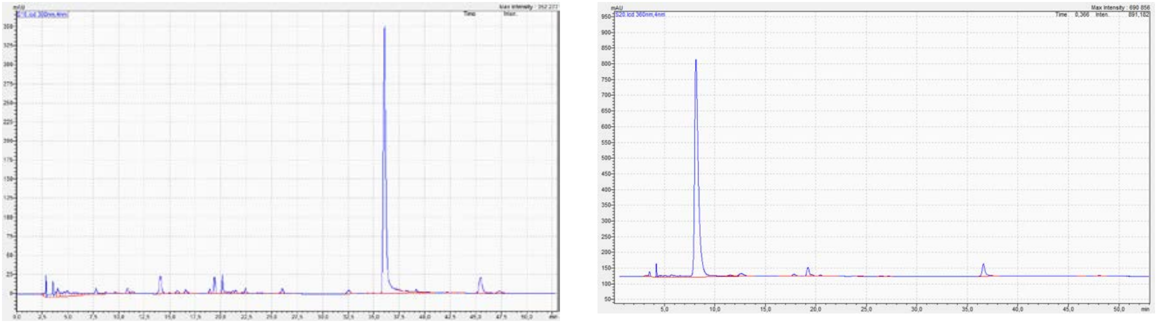

S10 **S20**

Figure S1. Fingerprint chromatograms of methanolic extracts of food supplements.

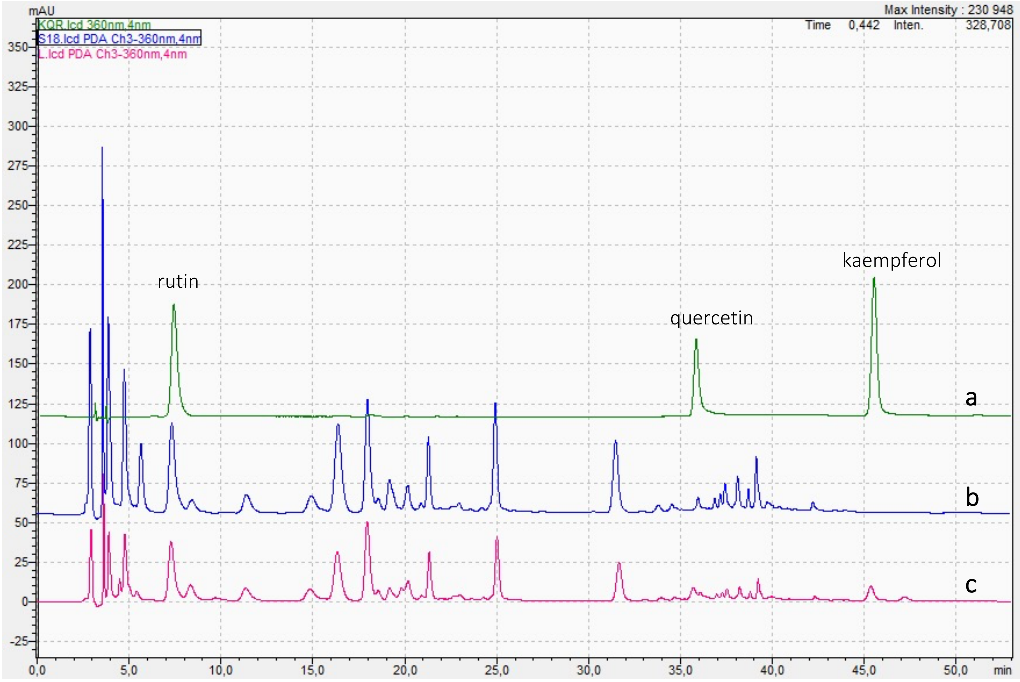

Figure S2. Chromatograms of flavonoids standard mixture (**a**), methanolic extract of food supplement S18 (**b**), and methanolic extract of ginkgo medicinal product L1 (**c**).

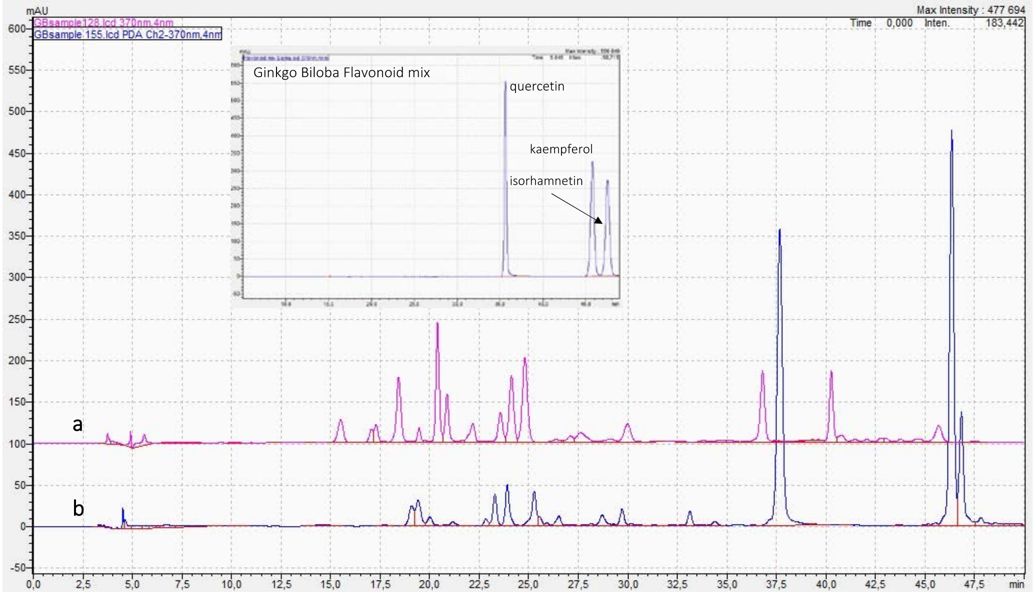

Figure S3. Chromatograms of *Ginkgo biloba* medicinal product before hydrolysis (**a**) and after acidic hydrolysis (**b**).

2. Antioxidant activity of 80% methanolic and agueous ectracts

Two portions of 100 mg of each food supplement were side-by-side extracted using 8 ml of water and 8 ml of 80% aqueous methanol solution. Samples were vortexed for 15 min and then placed into the ultrasonic bath for the next 15 min. DPPH• radical (2,2-Diphenyl-1-picrylhydrazyl) was used to assess the antiradical activity of extracts. The methanolic DPPH solution (0,05 mmol/L) was prepared and stored in darkness before the experiments. A volume of 150 µL non-hydrolyzed diluted methanolic or aqueous extract was mixed with 1ml DPPH• solution and incubated at a temperature of 25 °C in the dark. After 30 min, the absorbance of samples (A) at a wavelength of 517 nm was measured. The results were presented as a percent of DPPH• inhibition. The inhibition of DPPH• radical (%) was calculated as follows: $[(A_0-A)/A_0] \times 100\%$, where A_0 is the absorbance of DPPH• solution. Assays were performed at least in triplicate.

Separate determinations of DPPH radical scavenging activity were made for 80% methanol and water solutions of each tested product. Fig. 1 shows the absolute value of the difference between the percent of DPPH deactivation by 80% methanolic and aqueous extract of each sample. The choice of solvent significantly influences the content of compounds responsible for the free radical scavenging power of the extract. *Ginkgo biloba* L. standardized extracts are commonly prepared by solvent extraction such as water, methanol, ethanol, or acetone. European Pharmacopoeia recommends extraction of Ginkgo leaf powder with 60% acetone (m/m). Plant phenolic compounds are well soluble in water, methanol, ethanol, or acetone. In contrast, free aglycones such as quercetin or kaempferol are poorly soluble in water.

As shown from Fig. S4, the difference in antioxidant activity of water and methanol solutions (expressed as a percentage of DPPH• inhibition) for herbal medicines is within 2-6 (absolute value). Such a slight difference proves the comparable solubility of the sample in the water and methanol-water (80:20) mixture. We found a similar results for supplements S1, S5, S15, and S18, but it doesn't necessarily mean that these products contain ginkgo extract and are of good quality.

For other supplements, the differences in DPPH• inhibition of their methanolic and aqueous solutions are significant (above 20%), which means that the sample components responsible for the antioxidant activity (mainly flavonoids) dissolve much better in methanol. In the case of differences in DPPH• deactivation by methanol and water extract above 10%, may suggest that these products contain poorly water-soluble flavonols such as quercetin and kaempferol or significantly elevated content of rutin.

Food
supplement

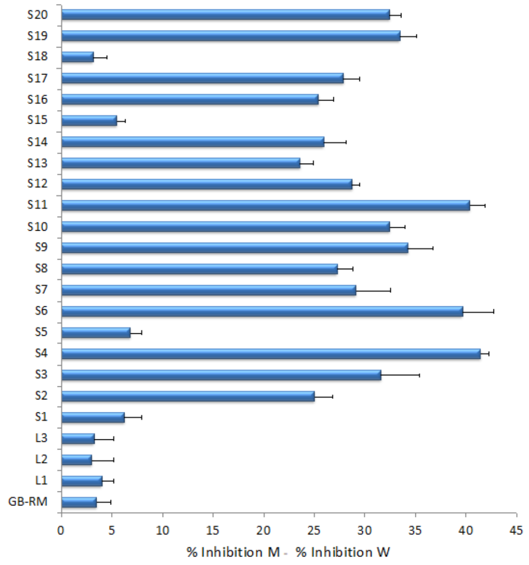

dard deviation

a **b**

Figure S4. The absolute difference between percentage inhibition of DPPH• solution by 80% methanol and aqueous extracts of ginkgo preparations (**a**), averaged % inhibition of DPPH• solution by 80% methanol and

aqueous extracts of ginkgo products. L1–L3 are medicinal products, S1–S20 food supplements, GB-XRM –*Ginkgo biloba* leaf extract reference material.

3. Two-trace two-dimensional correlation spectra

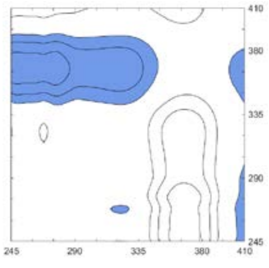

S2 **(A)**

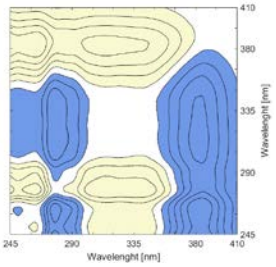

S2 **(B)**

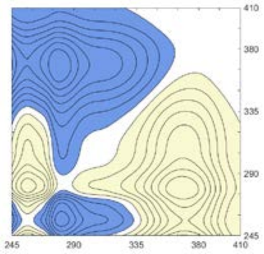

S2**(C)**

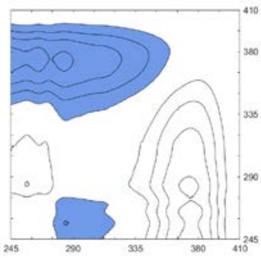

S3 **(A)**

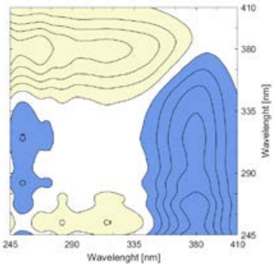

S3 **(B)**

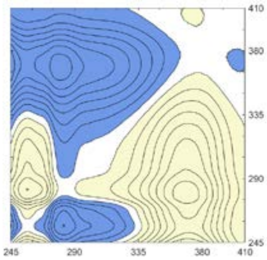

S3**(C)**

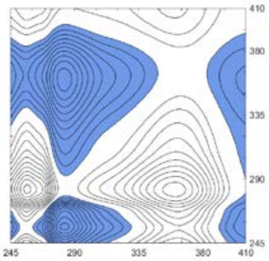

S4 **(A)**

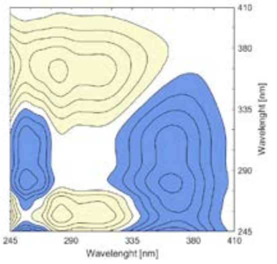

S4 **(B)**

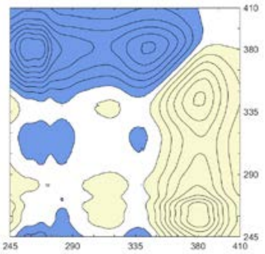

S4**(C)**

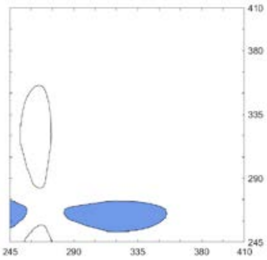

S5 **(A)**

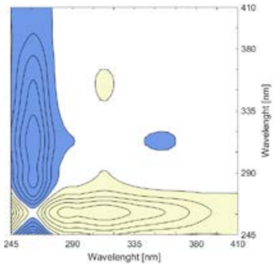

S5 **(B)**

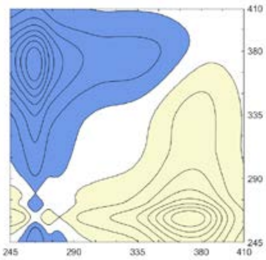

S5**(C)**

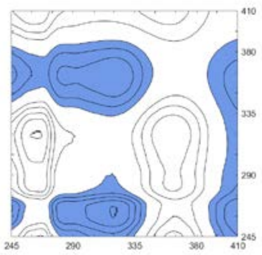

S6 **(A)**

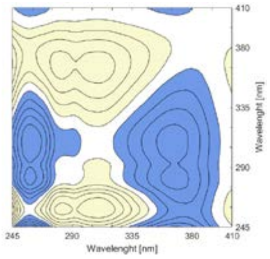

S6 **(B)**

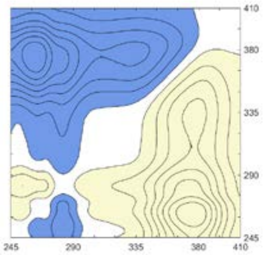

S6**(C)**

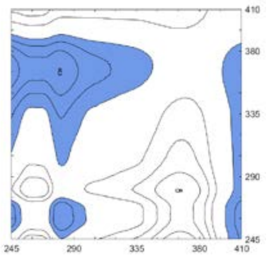

S7 **(A)**

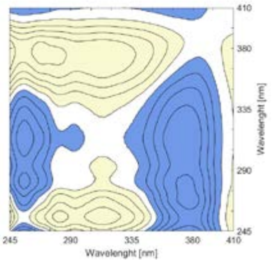

S7 **(B)**

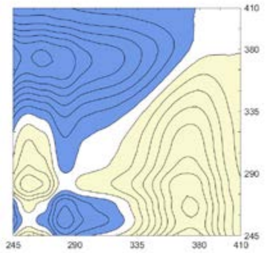

S7**(C)**

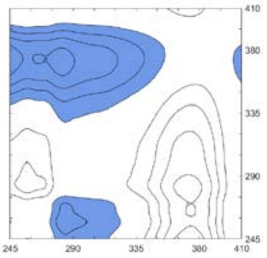

S8 **(A)**

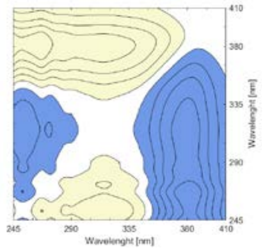

S8 **(B)**

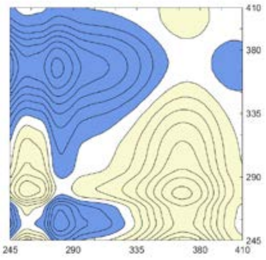

S8**(C)**

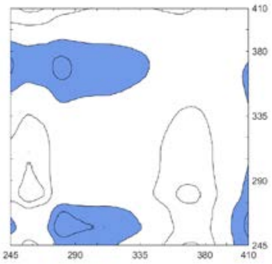

S9 **(A)**

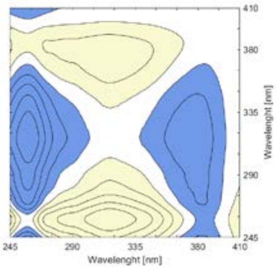

S9 **(B)**

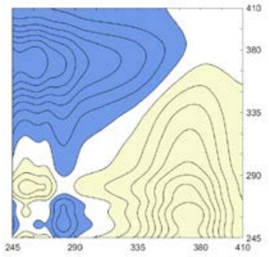

S9**(C)**

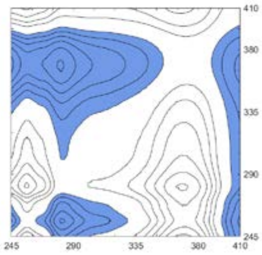

S10 **(A)**

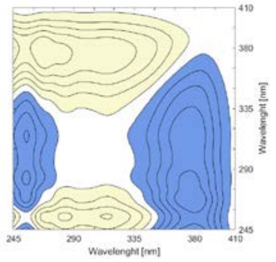

S10 **(B)**

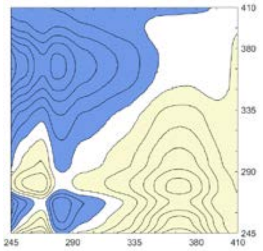

S10 **(C)**

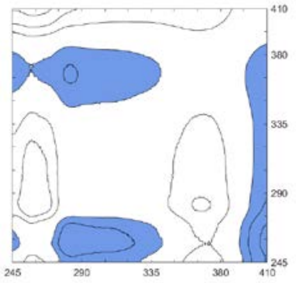

S12 **(A)**

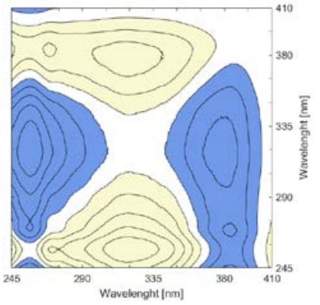

S12 **(B)**

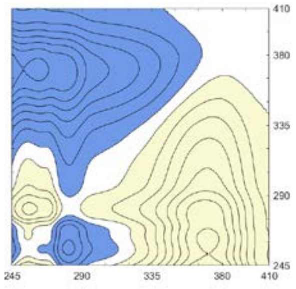

S12 **(C)**

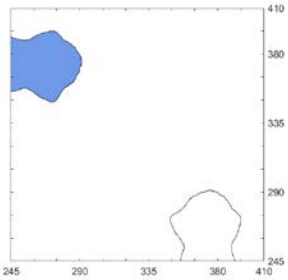

S13 **(A)**

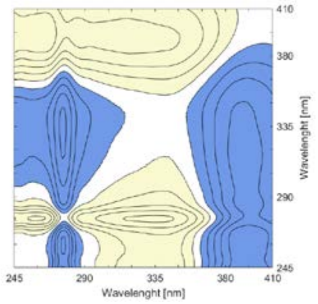

S13 **(B)**

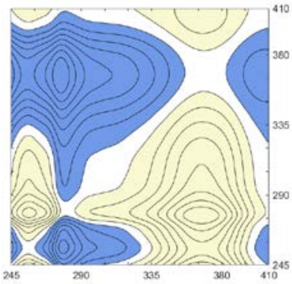

S13 **(C)**

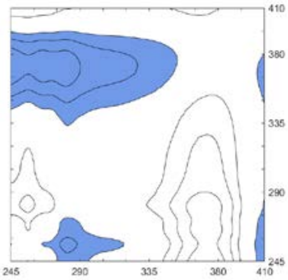

S14 **(A)**

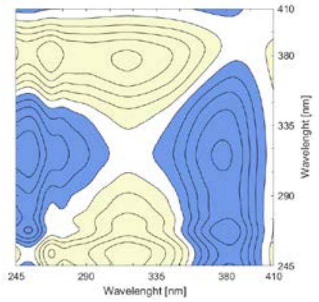

S14 **(B)**

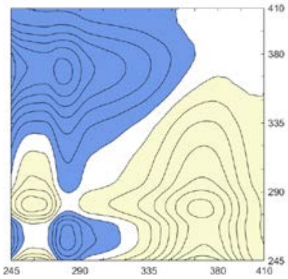

S14 **(C)**

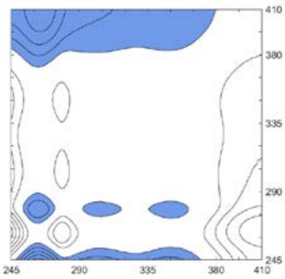

S16 **(A)**

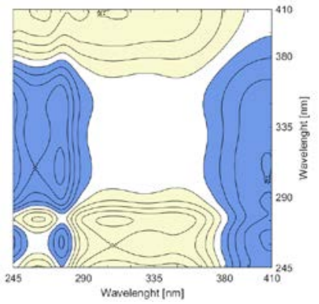

S16 **(B)**

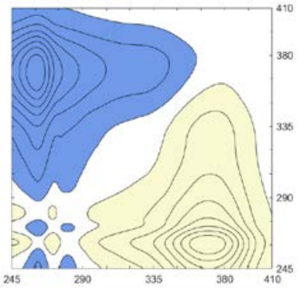

S16 **(C)**

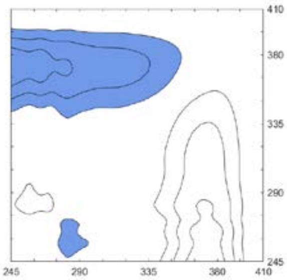

S17 **(A)**

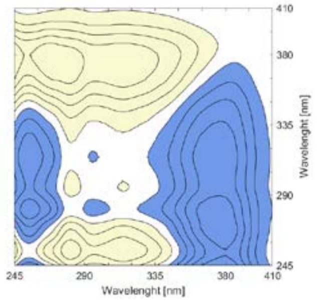

S17 **(B)**

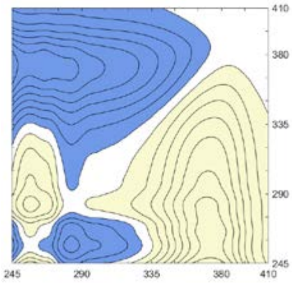

S17 **(C)**

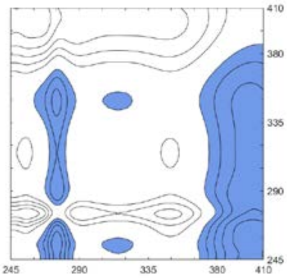

S18 **(A)**

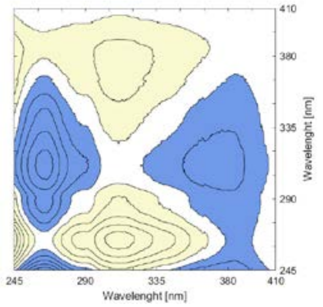

S18 **(B)**

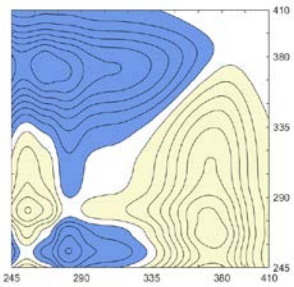

S18 **(C)**

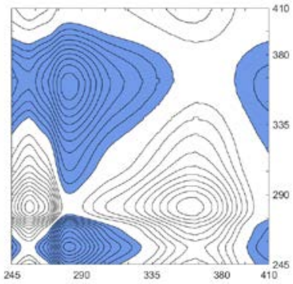

S19 **(A)**

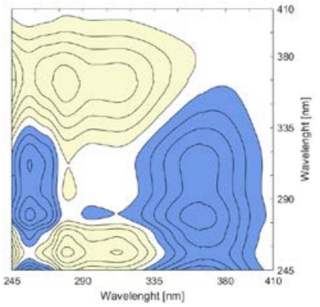

S19 **(B)**

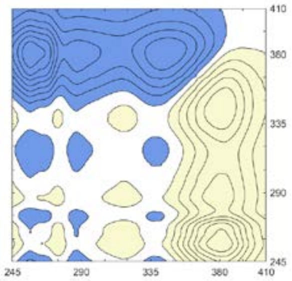

S19 **(C)**

Figure S5. Representative asynchronous 2T2D correlation spectra: **(A)** spectra of 80% methanol extract vs. water extract, **(B)** spectra of food supplements vs. spectrum of ginkgo XRM, **(C)** spectra of food supplements vs. averaged spectrum of adulterants: kaempferol, quercetin, and rutin.

Table S1. The spectral coordinates of characteristic cross peaks in Fig. 5 (s – sample spectrum, r – reference spectrum), + or – indicate positive or negative peaks.

**Kaempferol**	**Quercetin**	**Rutin**	**Ginkgo RM**
s(v_1)/r(v_2) [nm/nm]	s(v_1)/s(v_2) [nm/nm]	s(v_1)/r(v_2) [nm/nm]	r(v_1)/s(v_2) [nm/nm]
255/267 (+)	255/268 (–)	258/276 (–)	257/278 (+)
255/315 (+)	255/348 (–)	258/382 (–)	275/370 (–)
255/370 (+)	265/280 (+)	278/338 (+)	
340/370 (+)	265/387 (+)	345/380 (–)	
	275/348 (+)		
	355/387 (–)		

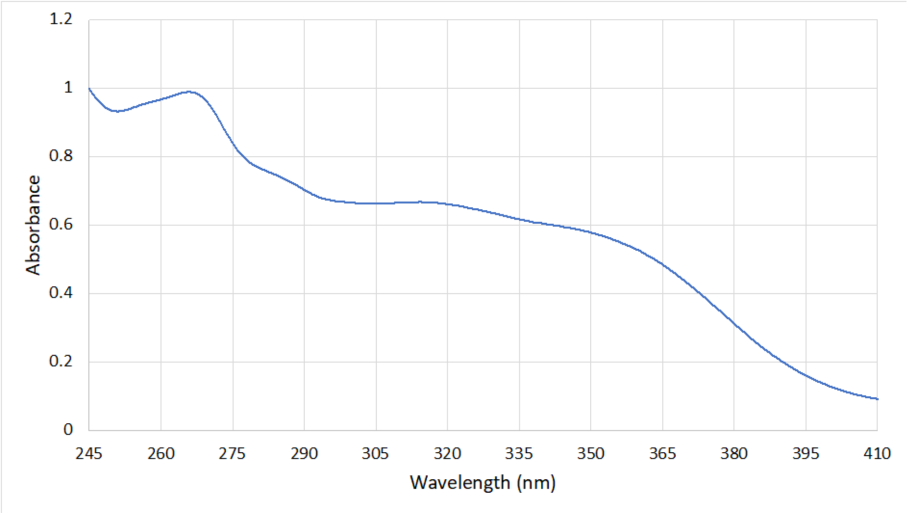

Figure S6. UV-Vis spectrum of Ginkgo biloba leaf extract (Reference Material) in 80% methanolic solution.

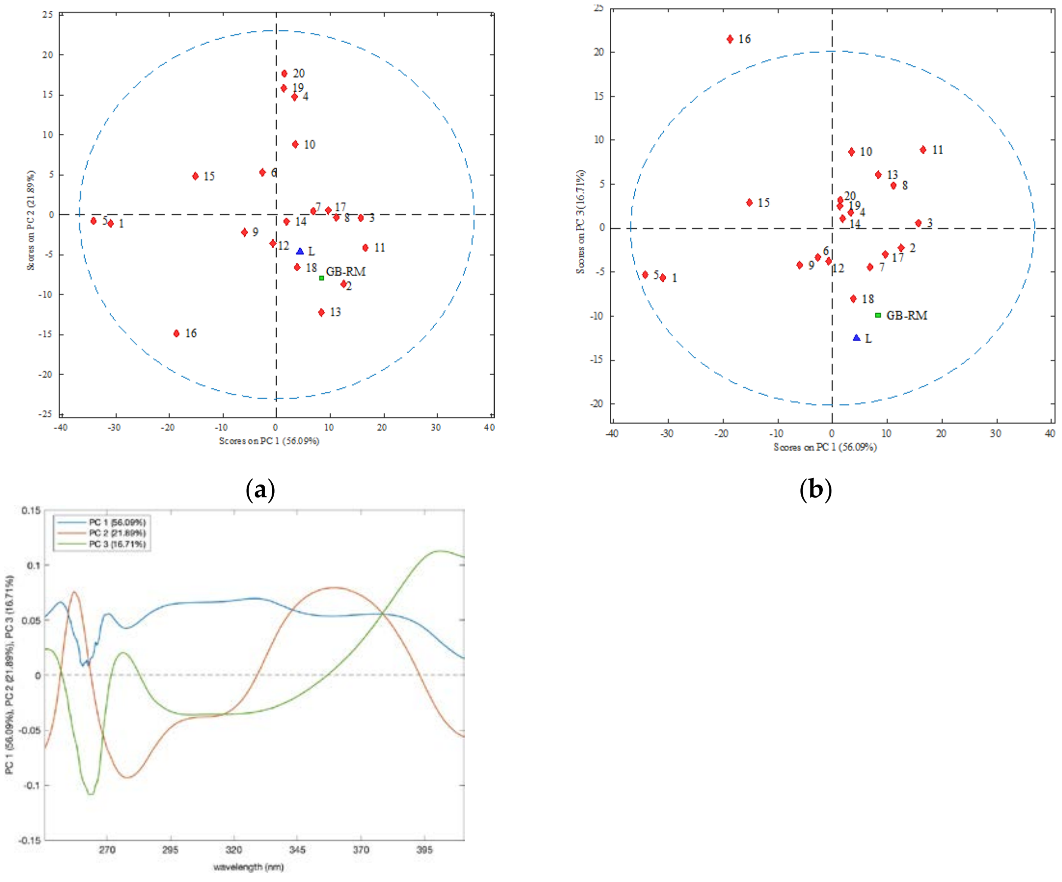

(**a**) (**b**)

(c)

Figure S7. PCA scores plots (**a**) and loadings plot (**b**) based on conventional 1D UV-Vis spectra of 80% methanolic extracts, RMSEC: 0.225; RMSECV: 0.596. L – ginkgo medicinal product, S1-S20 – food supplements, GB-RM – Ginkgo biloba leaf extract (Reference Material).

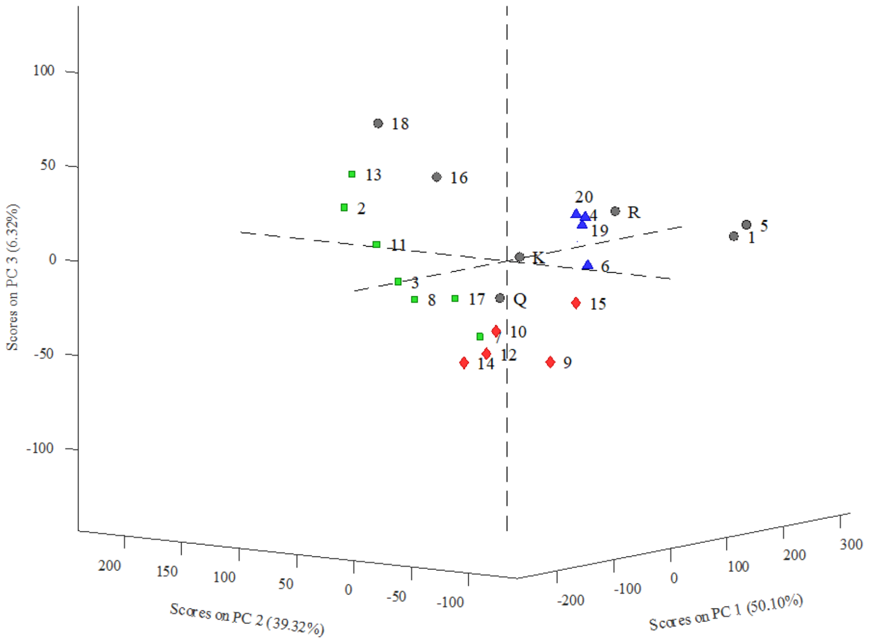

Figure S8. MPCA scores plot (PC1, PC2 and PC3) based on 2T2D correlation maps calculated for two spectra of 80% methanol extracts food supplements vs. ginkgo XRM as sample and reference spectrum, respectively. RMSEC: 0.081; RMSECV: 0.101. The blue triangles indicate supplements with rutin as main component, red diamonds – supplements with quercetin and green squares represent supplements with quercetin and kaempferol. Probability of confidence ellipse was 90%

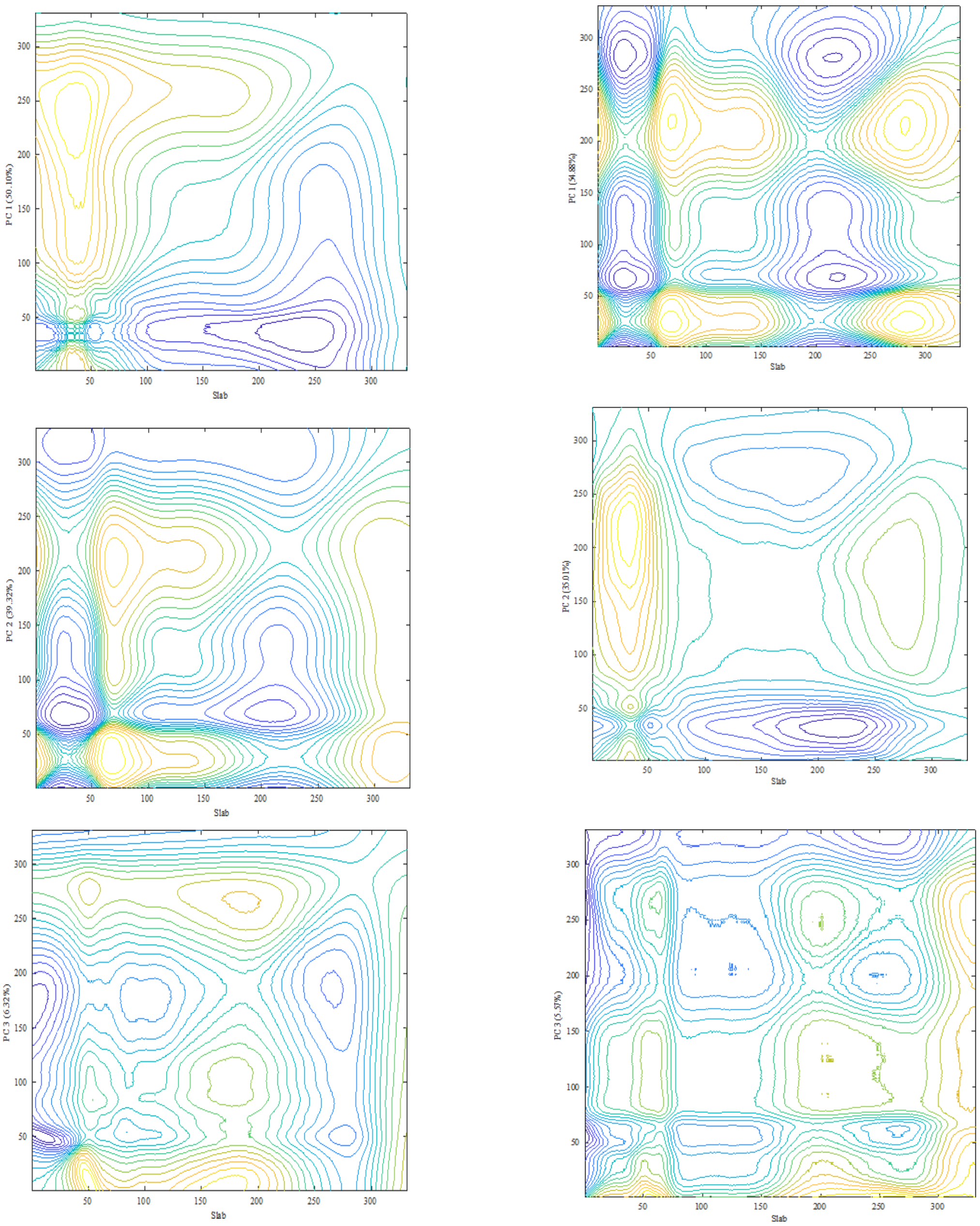

(**a**) (**b**)

Figure S9. Loadings plots for MPCA models based on spectra of series B (**a**) and series C (**b**).
